# *In vitro* evaluation of methylglyoxal as an antibacterial additive to bone cement

**DOI:** 10.3389/fbioe.2025.1661383

**Published:** 2025-09-10

**Authors:** Maja Charlotte Bohn, Hilke Oltmanns, Jessica Meißner

**Affiliations:** Department of Pharmacology, Toxicology and Pharmacy, University of Veterinary Medicine Hannover, Hanover, Germany

**Keywords:** methylglyoxal, periprosthetic joint infections, bone cement, antibacterial, biocompatibility

## Abstract

Periprosthetic joint infections (PJIs) are severe complications following surgical joint replacement and one of the main reasons for implant failure in human and veterinary medicine. Due to the global rise in antibiotic resistances and failure to prevent and treat PJIs, it is necessary to identify new antibacterial substances for the management of these infections. Methylglyoxal (MGO) is a dicarbonyl compound that has been identified as the main antibacterial component in Manuka honey. The aim of the study was to evaluate the suitability of MGO as an additive to polymethylmethacrylate bone cement in connection to PJIs. To test the antibacterial activity of pure MGO and MGO-containing bone cement against clinical isolates of *Staphylococcus (S.) pseudintermedius*, minimal inhibitory concentrations (MICs) were determined, growth of bacteria on bone cement was visualized, and the influence on infection of human osteosarcoma (HOS) cells was examined. Cytotoxicity of pure MGO and MGO-containing bone cement against HOS cells was analyzed with viability and proliferation assays, staining of cells on bone cement surface, and measurement of Interleukin-6 (IL-6) release. Activation of p38 MAP kinase was analyzed using Western blotting. MGO inhibited growth of *S. pseudintermedius* at 0.15 mg/mL, reduced bacterial colonization of bone cement at 25 mg per bone cement platelet, and reduced infection of HOS cells at 0.05 mg/mL. The IC_50_ of pure MGO for cell viability was 0.17 mg/mL. At higher concentrations, bone cement with MGO reduced viability and proliferation, but did not cause IL-6 release. Western blots revealed p38 activation following MGO treatment, indicating involvement of the p38 pathway in stress reactions due to the treatment. Taken together, effectiveness of MGO against PJI-relevant *S. pseudintermedius* could be shown but biocompatibility was limited and further research is necessary to enhance biocompatibility.

## 1 Introduction

Manuka honey, from the Manuka tree (*Leptospermum scoparium*) native to Australia and New Zealand (El-Senduny et al., 2021), is used in various medical applications due to its antibacterial properties. Formulations with Manuka honey are established as wound gels (Simon et al., 2006; Robson et al., 2009; Dryden et al., 2014; Harsent et al., 2022) and studies have shown that Manuka honey can act synergistically with selected antibiotic drugs, e.g., rifampicin (Jenkins and Cooper, 2012; Muller et al., 2013; Liu et al., 2017). New techniques including Manuka honey microneedle patches or coated wound dressings are established for the treatment of surgical site infections and wounds (Bulman et al., 2017; Frydman et al., 2020). While other medicinal honeys such as Revamil^®^ source honey provide antibacterial activity through molecules like bee defensin-1 and H_2_O_2_, antibacterial activity of Manuka honey can in parts be explained by methylglyoxal concentrations (Kwakman et al., 2011). Methylglyoxal (MGO), a dicarbonyl compound formed from dihydroxyacetone during honey maturation (Hossain et al., 2023), has been identified as one of the main antibacterial components in Manuka honey (Mavric et al., 2008). Growth inhibiting effects have been shown for several pathogens, including *Pseudomonas (P.) aeruginosa* (Hayashi et al., 2014), *Staphylococcus (S.) aureus* and *Escherichia coli* (Mavric et al., 2008). Furthermore, *in vitro* anti-biofilm activity has been shown for *Pseudomonas aeruginosa* and methicillin-resistant *Staphylococcus aureus* (Kilty et al., 2011). Different mechanisms of antibacterial action have been proposed for MGO, mainly based on the binding of proteins, lipids, and nucleic acids. Binding these macromolecules leads to the formation of advanced glycation end-products (AGEs) and damage of their function (Schalkwijk and Stehouwer, 2020). In bacteria, MGO can bind fimbriae and flagellar proteins and disrupt their structural integrity and function, so that bacterial adherence and motility is limited (Rabie et al., 2016). Therefore, MGO can act both bactericidally by killing bacteria through AGE formation (Roberts et al., 2012) and bacteriostatically by inhibiting motility and attenuating virulence (Rabie et al., 2016; Anaya-Sanchez et al., 2025). Next to treatment with MGO only, MGO has been shown to act synergistically with antibiotics. MGO could increase sensitivity of *S. aureus* to linezolid (Hayes et al., 2018) and acted synergistically with piperacillin, amikacin and carbenicillin against *P. aeruginosa* (Mukherjee et al., 2011). MGO is also produced in mammalian cells as a by-product of glycolysis and is elevated in diabetic conditions. Through AGE formation, it contributes to diabetic complications like neuropathy, nephropathy or impaired wound healing (Schalkwijk and Stehouwer, 2020; Seto et al., 2025). As an antimicrobial effector, Manuka honey-derived MGO contributes to the activation of mucosal-associated invariant T cells (MAIT cells), which protect from bacterial pathogens after recognition of a bacterial metabolite (Tang et al., 2020).

Total joint arthroplasty is a common procedure to replace destructive joints. More than 340,000 primary knee and hip arthroplasties were conducted in Germany in 2023 (Grimberg et al., 2024). Next to fractures and aseptic loosening of the implant due to an immune reaction to implant particles, periprosthetic joint infection (PJI) is the major complication after total joint arthroplasty (Grimberg et al., 2024; Piuzzi et al., 2024). Consequently, PJI can lead to implant failure, revision surgery or even death of the patient (Gehrke et al., 2024). PJIs can occur directly after surgery or weeks later through different routes of infection (Benito et al., 2019). In veterinary medicine, total joint replacement is mainly established as hip, knee, and elbow replacements in small animals (Allen, 2012). The postoperative infection rate in small animals ranges from 2.6%–10% (Weese, 2008). Both virulent and opportunistic pathogens serve as causative agents for PJIs. In humans, common virulent pathogens are *Staphylococcus aureus* (Benito et al., 2019; Gatti et al., 2022), *Enterococcus* (Renz et al., 2019; Thompson et al., 2019), *Streptococcus* and *Bacillus* species (Bemer et al., 2014). Coagulase-negative Staphylococci often display as opportunistic pathogens (Benito et al., 2019; Gatti et al., 2022). In small animals, more than 50% of postoperative infections are caused by *S. aureus* and *Staphylococcus pseudintermedius* (Hayes et al., 2013). *S. pseudintermedius* is a coagulase-positive opportunistic pathogen colonizing mainly cats and dogs. It causes skin infections in these species (Devriese et al., 2005; Perreten et al., 2010), but can also act as a zoonotic pathogen and infect humans (Van Hoovels et al., 2006), especially immunocompromised patients and humans in close contact with companion animals (Roberts et al., 2024; Vines et al., 2024). Methicillin-resistant *S. pseudintermedius* (MRSP) are an increasing challenge, with lineages spread over Europe and North America (Perreten et al., 2010) and methicillin resistances often connected to multidrug resistances (Myrenas et al., 2024). Colonization with MRSP has been confirmed in veterinary employees and in the environment of a small animal hospital (Fessler et al., 2018). Antibiotic resistance in general is a global threat for human and veterinary health. Resistance frequently occurs in places with high antibiotic consumption, e.g., hospitals, agriculture, or the environment (Almagor et al., 2018; Skandalis et al., 2021).

Polymethylmethacrylate (PMMA) bone cement is widely used for orthopedic treatments. While the main function is the fastening of joint implants, PMMA bone cement is also used to restore fractured bones or to stabilize vertebrae in atlantoaxial instability (Hamajima et al., 2020; Tabanez et al., 2021). Bone cement is also established for local delivery of antibiotic drugs in endoprosthetics and as beads on a string for the use directly at the site of infection (Hayes et al., 2013). Most antibiotics used in bone cement are aminoglycosides and aminopeptides (Cyphert et al., 2018). Such antibiotic-laden bone cements (ALBCs) are commercially available and have the advantage that high drug concentrations at the site of infection can be reached (Shen et al., 2019). However, the effectiveness of ALBCs has been discussed critically due to limited evidence (Hoskins et al., 2020; Sebastian et al., 2020). An *in vitro* study has shown that the combination of two antibiotics in ALBC is more effective against gram-negative pathogens than the use of just one antibiotic drug (Cara et al., 2022). For active substances that are added to bone cement it applies that the quantity of substance released from the cement has to be above the minimal inhibitory concentration (MIC) and the minimal bactericidal concentration (MBC) of the pathogen (Kühn, 2014). Requirements for the choice of a suitable active substance are that the substance is water-soluble and stable during PMMA polymerization (Carli et al., 2018). Substance is released mostly from the surface of the bone cement, following the law of diffusion (Anagnostakos and Meyer, 2017; Sun et al., 2021). Release proceeds in two phases with a first “burst” phase in which high substance concentrations are reached and the second phase with stable release of low concentrations (Sun et al., 2021). Due to this release profile, it is critically discussed if low release of antibiotic drugs from ALBC in the later phase after surgery could lead to increased occurrence of new resistances. According to data from the National Joint Registry database for England and Wales, using gentamicin-loaded bone cement in primary surgery led to a higher proportion of gentamicin-resistant strains in PJIs (Holleyman et al., 2019). Similar results were observed for local clindamycin delivery in a clinical study (Tyas et al., 2018). However, other studies with retrospective analyses of PJI cases could not find an increased risk of antibiotic resistance after the use of ALBC (Schmitt et al., 2020; Tootsi et al., 2022).

With the global challenge of antimicrobial resistances, it is necessary to identify new antimicrobial substances also for the management of PJIs. These new substances must be effective against relevant pathogens and show high biocompatibility at the same time. The aim of this study was therefore to provide an evaluation of MGO together with bone cement as a prevention and treatment option against PJIs caused by *S. pseudintermedius*.

## 2 Materials and methods

### 2.1 Bone cement

PALACOS^®^ LV bone cement (Heraeus Medical, Wehrheim, Germany) was mixed under sterile conditions and filled into silicone molds to create round platelets with a diameter of 9 mm (height 4 mm, volume 254.47 mm^3^). MGO solution (33.4 wt% in water; Thermo Fisher Scientific, Waltham, United States) was added to the liquid component of the bone cement before mixing the cement components.

### 2.2 Cell line and culture media

For determination of cytotoxicity, bacterial adhesion and internalization, and preparation of cell lysates for Western blot, the human osteosarcoma cell line HOS (CRL-1543), obtained from the American Type Culture Collection (ATCC, Manassas, United States), was used. Cells were subcultured three times a week and kept in Eagle’s Minimum Essential Medium with Earle’s balanced salts (EMEM; Carl Roth, Karlsruhe, Germany) with 10% fetal bovine serum (FBS; Bio & Sell, Feucht, Germany), 1% l-glutamine (GIBCO, Paisley, United Kingdom), 1% non-essential amino acids (Carl Roth, Karlsruhe, Germany) and 1% penicillin/streptomycin (GIBCO, Grand Island, United States) in an incubator at 37 °C and 5% CO_2_.

### 2.3 Bacterial strains

For microbiology experiments, one sensible (SP1) isolate of *S. pseudintermedius* and two multiresistant isolates (RSP1 and RSP2) were used. Isolates were taken from dogs in the Clinic for Small Animals and identified with matrix-assisted laser desorption ionization-time of flight mass spectrometry (MALDI-TOF MS) and assessed for resistances in the Institute for Microbiology, both located at the University of Veterinary Medicine Hannover, Germany. Bacterial stocks were kept in cryomedium with 80% glycerin at −80 °C and were subcultured on Columbia agar containing 5% sheep blood (Oxoid Deutschland, Wesel, Germany) at 37 °C overnight prior to the experiments. During experiments, bacteria were cultured in Mueller Hinton bouillon (MHB; Sifin Diagnostics, Berlin, Germany) at 37 °C.

### 2.4 Antibacterial activity testing

Minimal inhibitory concentrations (MICs) of MGO for the three clinical isolates of *S. pseudintermedius* were determined with broth microdilutions according to CLSI (CLSI, 2012). MGO was diluted in MHB to concentrations of 0.025 mg/mL to 0.78 mg/mL and a bacterial suspension with a turbidity of 0.5 McFarland units was prepared in 0.9% NaCl solution (B. Braun, Melsungen, Germany). MGO solution and bacterial suspension were added to a U-bottom 96-well microtiter plate along with a negative control with MHB and sterile NaCl only and a growth control without MGO. Inhibition of bacterial growth was evaluated after 18 h of incubation through optical screening for bacterial pellets.

For analysis of bacterial growth on bone cement with MGO, bone cement platelets containing 5 mg, 10 mg or 25 mg MGO per platelet or without additive (control) were fastened on the inside of the lid of a 24-well culture plate and hung into *S. pseudintermedius* RSP1 suspended in MHB. The construct was incubated for 72 h at 37 °C. Staining with the LIVE/DEAD™ BacLight™ Bacterial Viability Kit (Thermo Fisher Scientific, Waltham, United States) according to the manufacturer’s instructions was conducted after the 72 h incubation. Live and dead bacterial cells were then examined by fluorescence microscopy (BZ-X800 Inverted Fluorescence-Phase Contrast-Microscope, Keyence Corporation, Osaka, Japan) at 470 nm and 545 nm excitation and 525 nm and 605 nm emission, respectively.

### 2.5 Influence of MGO on bacterial adhesion and internalization into HOS cells

HOS cells were seeded into 24-well culture plates at 40,000 cells per well and grown to confluency in antibiotic-free cell culture medium. A suspension of the susceptible isolate SP1 was prepared in 0.9% sterile sodium chloride solution to a density of 0.5 McFarland units and diluted to create a MOI (multiplicity of infection) of 10:1. HOS cells and SP1 were co-incubated together with 0.05 mg/mL MGO or cell culture medium only for two or 4 hours. After co-incubation, cells were washed three times with sterile phosphate-buffered saline (PBS) and then incubated with cell culture medium containing 100 μg/mL gentamicin for 2 hours to kill extracellular bacteria or incubated with antibiotic-free cell culture medium to keep adherent bacteria. HOS cells were washed again and then scraped off the well bottom, collected, centrifuged, and lysed by osmotic shock by adding bi-distilled water. Lysis was aided by pipetting the cell suspension up and down with syringe and needle. Lysates were diluted in PBS, plated onto Columbia agar containing 5% sheep blood (Oxoid Deutschland, Wesel, Germany) and incubated for 24 h at 37 °C. Colony forming units in the lysates were determined by counting colonies.

### 2.6 Measurement of cell viability and proliferation

Cytotoxic concentrations of MGO were determined measuring the viability and proliferation of HOS cells. For that, several concentrations of MGO diluted in cell culture medium were prepared and cells were treated with the dilutions for 24 h. Cells were grown to confluency prior to viability testing or were seeded 4 hours prior to proliferation testing. Viability was measured using MTS assay and proliferation was tested via crystal violet assay. Cytotoxicity of bone cement was tested by measuring cell viability and proliferation after treatment with bone cement supernatants. To create supernatants, bone cement platelets with 5 mg MGO, 10 mg MGO, 25 mg MGO, or without additive (control) were incubated in 1, 3, 10 or 30 mL cell culture medium for 24 h. HOS cells were treated (grown to confluency for viability testing, 4 hours after seeding for proliferation testing) with the supernatants, 1 μg/mL lipopolysaccharide (LPS) from *E. coli* O55:B5, and 50 μg/mL peptidoglycan (PGN) (as positive controls for Enzyme-linked Immunosorbent Assay (ELISA)) for 24 h. After treatment, the cell supernatants were collected for ELISAs and both cell viability and proliferation were measured. Growth of HOS cells on the surface of bone cement platelets with MGO was analyzed by seeding cells onto the platelets, incubating for 24 h and staining the cells with the Live/Dead™ Viability/Cytotoxicity Kit (Thermo Fisher Scientific, Waltham, United States) according to the manufacturer’s protocol. Live and dead cells were visualized with fluorescence microscopy (BZ-X800 Inverted Fluorescence-Phase contrast-Microscope, Keyence Corporation, Osaka, Japan) at 470 nm and 545 nm excitation and 525 nm and 605 nm emission, respectively. Ten sections of every platelet with a size of 2.2 mm^2^ each were photographed and cell numbers in each photo were counted with the Keyence BZ-X800 Analyzer software (Keyence Corporation, Osaka, Japan). The mean number of living or dead cells in the ten sections of every platelet and the cell number per mm^2^ were calculated.

### 2.7 Enzyme-linked immunosorbent assay (ELISA)

Release of Interleukin-6 (IL-6) and Interleukin-1β (IL-1β) in the samples from HOS cells incubated with bone cement supernatants was measured with DuoSet^®^ ELISA kit for human IL-6 (R&D Systems, Minneapolis, United States) and DuoSet^®^ ELISA kit for human IL-1β/IL-1F2 (R&D Systems, Minneapolis, United States), respectively. ELISA was performed according to the manufacturer’s instructions. Optical density of samples and standards was measured at 450 and 570 nm with the Multiskan FC microplate reader (Thermo Fisher Scientific, Waltham, United States). Cytokine concentration was calculated with a standard curve generated with Microsoft Excel 365.

### 2.8 Western blot

HOS cells were treated for 30 min with 0.15, 0.25, or 0.5 mg/mL MGO or bone cement with 5 mg MGO, 10 mg MGO, 25 mg MGO or without additive incubated in 3 mL medium. After the treatment, cells were washed twice with ice-cold PBS and lysed in radioimmunoprecipitation assay (RIPA) buffer containing protease (cOmplete™ ultra mini EDTA free, Roche, Mannheim, Germany) and phosphatase inhibitors (PhosSTOP™, Roche, Mannheim, Germany). Lysates were centrifuged at 15,000 x g and 4 °C and the protein-containing supernatants were taken and stored at −80 °C until use. Total protein content in all supernatants was determined using Pierce™ BCA Protein Assay Kit (Thermo Scientific, Rockford, United States). Based on the results of this assay, sample aliquots were adjusted to 10 µg protein per 10 µL and mixed with Laemmli sample buffer (with 25% glycerol (Carl Roth, Karlsruhe, Germany), 5% sodium dodecyl sulfate, 5% β-mercaptoethanol (Sigma-Aldrich Chemie, Steinheim, Germany), 150 mM Tris pH 6.8, 0.05% bromophenol blue (Merck KGaA, Darmstadt, Germany). Proteins were separated using 10% Mini-PROTEAN TGX Precast Protein gels (Bio-Rad, Feldkirchen, Germany) and then blotted onto nitrocellulose membranes (Cytiva Amersham™, Little Chalfont, United Kingdom). Total protein on the membranes was stained with Ponceau S Red (Carl Roth, Karlsruhe, Germany; 0.01% in 1% acetic acid) and the total protein image was taken (ChemiDoc MP Imaging System, Bio-Rad, Feldkirchen, Germany) before destaining the membranes with Tris-buffered saline with 1% Tween-20 (TBS-T) and blocking in nonfat milk for 2 h at room temperature. Membranes were washed with TBS-T after each incubation step. Primary antibodies used were p38 MAPK Polyclonal Antibody (AHO1202, Thermo Fisher Scientific, Waltham, United States), 1:1000 and phospho-p38 MAPK (Thr180, Tyr182) Polyclonal Antibody (44-684G, Thermo Fisher Scientific, Waltham, United States) 1:1000 in 5% BSA in TBS-T. The secondary antibody used was Goat-anti rabbit IgG (H + L) Secondary Antibody, HRP conjugated (31,466, Thermo Fisher Scientific, Waltham, United States) 1:20,000 in nonfat milk. For the detection of protein bands, membranes were incubated with SuperSignal West Pico PLUS chemiluminescence substrate (Thermo Fisher Scientific, Waltham, United States) and imaged with the ChemiDoc MP Imaging System. Total protein normalization and image analysis was carried out using ImageLab software (Bio-Rad, Feldkirchen, Germany).

### 2.9 Statistical analysis

Statistical analysis was conducted with GraphPad Prism 10 (GraphPad Software, Boston, United States). Non-parametric data was assessed for statistical significance with Kruskal–Wallis tests with Dunn’s multiple comparison, Friedman test, or Mann-Whitney test. Results with p-values ≤0.05 were considered statistically significant: *p ≤ 0.05, **p ≤ 0.01, ***p ≤ 0.001. Analyzed data include at least six biological replicates.

## 3 Results

### 3.1 Antibacterial activity

The minimal inhibitory concentration (MIC) of MGO against one sensible (SP1) and two multi-resistant (RSP1 and RSP2) strains of *S. pseudintermedius* was 0.15 mg/mL for all three isolates.

**FIGURE 1:**
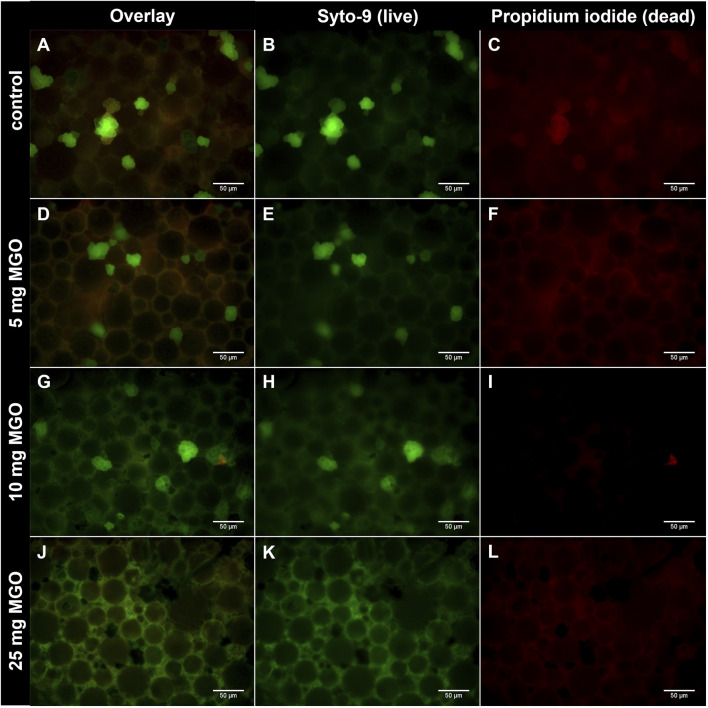
Growth of *S. pseudintermedius* RSP1 on bone cement platelets with MGO. *S. pseudintermedius* were stained with Syto-9 (green) to visualize live bacteria and with propidium iodide (red) to visualize dead bacteria. **(A**–**C)** Bone cement without additive (control). Grouped bacteria are attached to the bone cement surface. **(D**–**F)** Bone cement with 5 mg MGO. **(G**–**I)** Bone cement with 10 mg MGO. **(J**–**L)** Bone cement with 25 mg MGO. No grouped bacteria are attached to the bone cement surface. Images of Syto-9 (**(B)**, **(E)**, **(H)**, **(K)**) and propidium iodide staining (**(C)**, **(F)**, **(I)**, **(L)**) as well as the composite image of both (Overlay; **(A)**, **(D)**, **(G)**, **(J)**) are shown for each MGO concentration. Scale bar: 50 µm.

To reach antibacterial concentrations in bone cement, 5 mg, 10 mg, and 25 mg MGO per platelet were chosen for incorporation. Attachment and growth of *S. pseudintermedius* RSP1 on the bone cement surface was analyzed (Figure 1). After 72 h of incubation, no continuous biofilm was observed; instead, bacteria appeared as separate groups on the bone cement. On bone cement without additives (Figures 1A–C), several groups of live bacteria (stained in green) and one group with live and dead bacteria (stained green and red) can be seen. Addition of 5 mg MGO to bone cement (Figures 1D–F) did not reduce attachment and growth of bacteria compared to control. Between the round bone cement particles, a slight red background staining was visible (Figure 1F). On bone cement with 10 mg MGO (Figures 1G–I), several groups of live bacteria and one group of dead bacteria were observed, so that there was no reduction of bacterial growth compared to the control. Between the cement particles green background fluorescence was visible. With 25 mg MGO incorporated (Figures 1J–L), no bacterial groups were attached to the bone cement surface, only green background fluorescence could be observed. Background fluorescence in general got more intense with rising MGO concentration in the bone cement (Supplementary Figure). To sum up, attachment and growth on the bone cement surface was only prevented by addition of 25 mg MGO.

### 3.2 Influence on bacterial adherence and internalization into HOS cells

**FIGURE 2:**
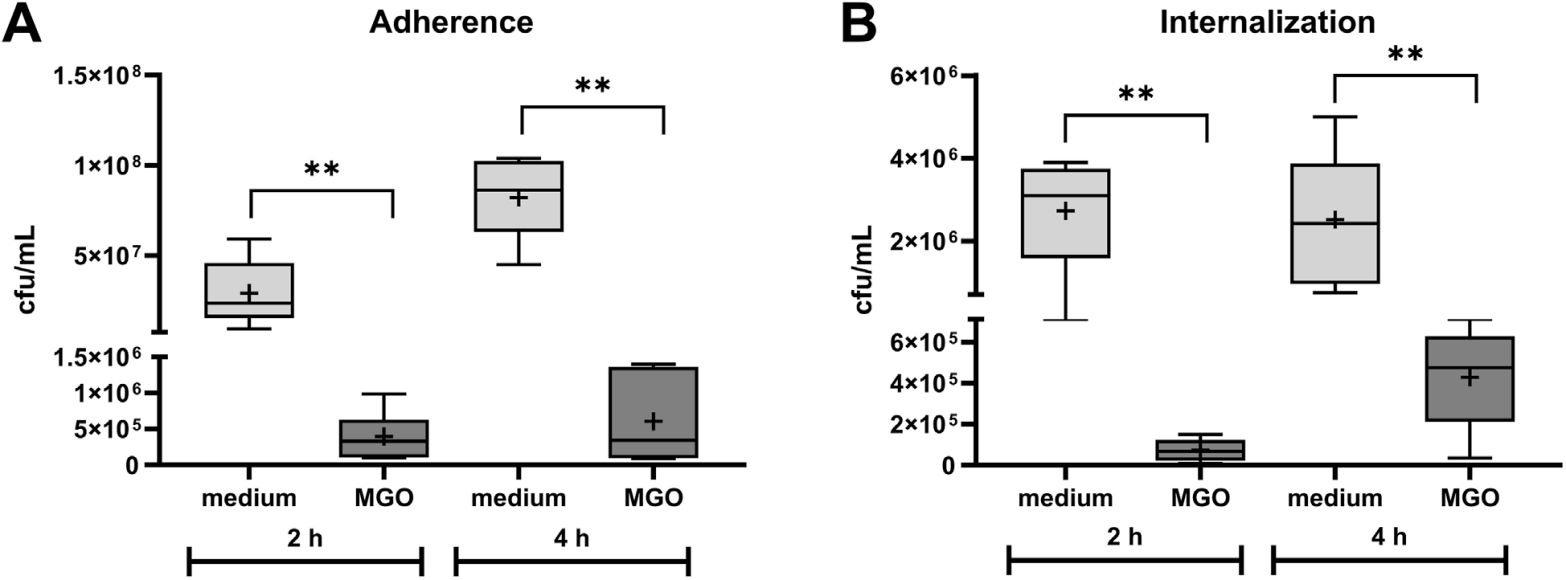
Influence of 0.05 mg/mL MGO on adherence **(A)** and internalization **(B)** of *S. pseudintermedius* SP1 to/into HOS cells. Data are shown as boxplots with min/max-whiskers with the mean depicted as + (n = 6 each). Adherent and internalized bacteria are quantified as colony forming units (cfu) per mL after 2 h and 4 h co-incubation of HOS cells, SP1, and MGO or cell culture medium. Mann-Whitney test; *p ≤ 0.05, **p ≤ 0.01; asterisks indicate significant differences between incubation with medium or MGO.

Adherence of *S. pseudintermedius* SP1 to HOS cells and internalization into the cells in the presence of 0.05 mg/mL MGO was compared to adherence and internalization without MGO (Figure 2). The count of adherent bacteria after two and 4 hours of infection was significantly lower in samples co-incubated with MGO (Figure 2A) and the difference between the groups was larger after 4 hours of infection. The count of internalized bacteria was significantly lower in the presence of MGO and the difference between MGO and medium was larger after 2 hours of infection (Figure 2B). Therefore, 0.05 mg/mL MGO reduced the infection of HOS cells.

### 3.3 Cell viability, proliferation, and cytokine release

**FIGURE 3:**
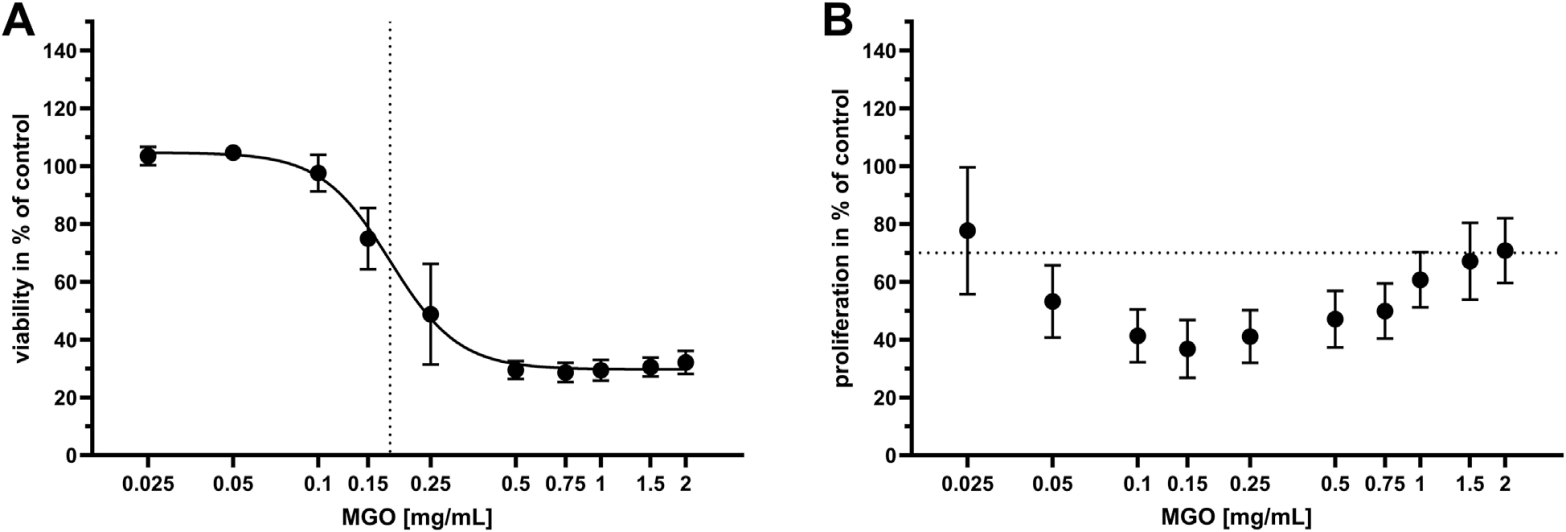
Viability and proliferation of HOS cells after treatment with different concentrations of MGO. Every value is the mean of at least six passages of HOS cells ±SD. Treatment with cell culture medium only served as the control. **(A)** Dose-response graph of 0–2 mg/mL MGO for viability of HOS cells. The dotted vertical line indicates the half-maximal inhibitory concentration (IC_50_), which is 0.17 mg/mL MGO. **(B)** Dose-response graph of 0–2 mg/mL MGO for proliferation of HOS cells. The dotted horizontal line indicates the 70% limit for cytotoxicity testing according to ISO 10993-5; 2009-06 (International Organization for Standardization, 2009).

To identify cytotoxic concentrations of MGO, the influence of 0–2 mg/mL MGO on viability and proliferation of HOS cells was examined. The results are displayed in dose-response graphs as percent of control (Figure 3). Cell viability (Figure 3A) was reduced at 0.15 mg/mL MGO and higher concentrations, with the half-maximal inhibitory concentration (IC_50_) at 0.17 mg/mL MGO. Proliferation of HOS cells (Figure 3B) was below 70% of control at 0.05–1.5 mg/mL, referring to the 70% limit for cytotoxicity testing according to ISO 10993-5; 2009-06 (International Organization for Standardization, 2009). At 0.025 mg/mL, the lowest tested MGO concentration, mean proliferation was at 80% of control, but the standard deviation indicates that this concentration caused cytotoxicity in some of the experiments. At the highest tested concentration, 2 mg/mL MGO, the mean proliferation was 72% of control. In general, the dose-response curve was U-shaped with the lowest proliferation at 0.15 mg/mL (Figure 3B).

**FIGURE 4:**
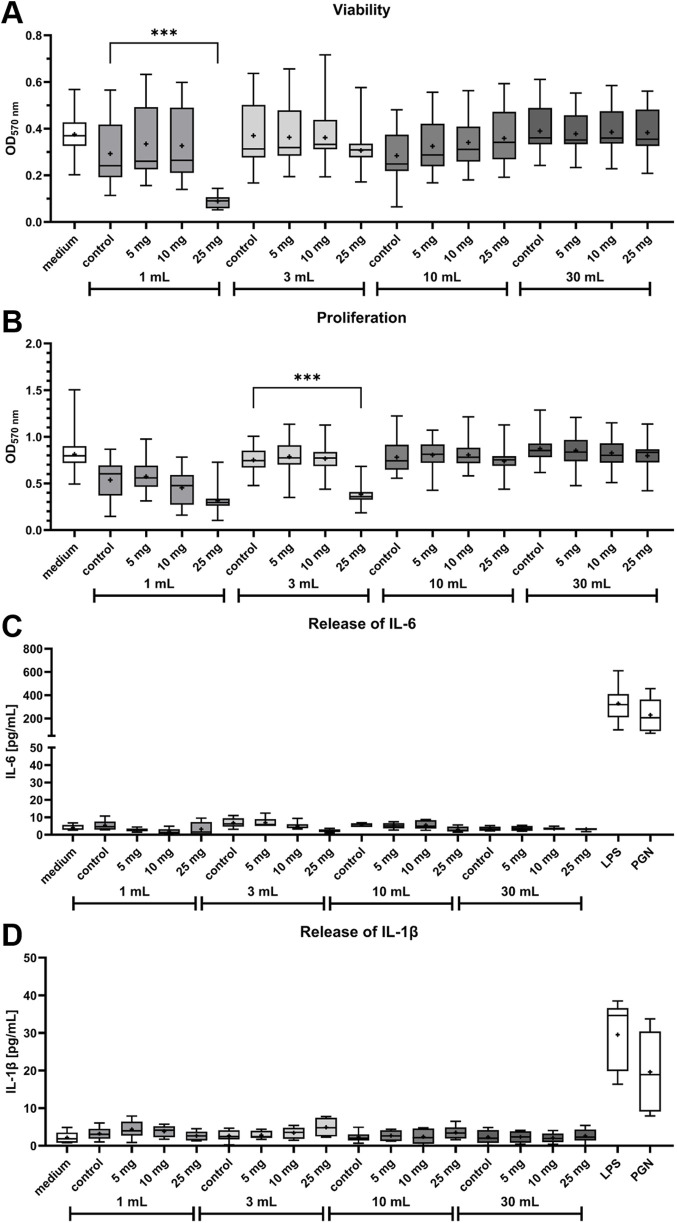
Influence of bone cement with MGO incubated in four different volumes of cell culture medium on viability, proliferation, release of Interleukin-6 (IL-6) and release of Interleukin-1β (IL-1β) of HOS cells. **(A,B)** Viability **(A)** and proliferation **(B)** of HOS cells after 24 h of treatment with supernatants of bone cement without additive (control), 5 mg MGO, 10 mg MGO, or 25 mg MGO. Data is shown as boxplots with min/max-whiskers and the mean depicted as + (n = 6). Kruskal–Wallis test with Dunn’s multiple comparisons test; *p ≤ 0.05, **p ≤ 0.01, ***p ≤ 0.001; asterisks indicate significant differences in comparisons to the samples’ respective control. **(C,D)** Release of IL-6 **(C)** and IL-1β **(D)** from HOS cells treated with supernatants of bone cement without additive (control), 5 mg MGO, 10 mg MGO, 25 mg MGO, 1 μg/mL lipopolysaccharide (LPS), or 50 μg/mL peptidoglycan (PGN) for 24 h. Treatment with LPS and PGN served as positive control. Data are shown as boxplots with min/max-whiskers and the mean depicted as + (n = 6). No significant differences were determined between cells treated with MGO-containing bone cement supernatants and the control.

Viability, proliferation and IL-6 release in HOS cells after treatment with bone cement supernatants were measured to test cytotoxicity of MGO together with bone cement. Viability of HOS cells was significantly reduced after treatment with supernatant of bone cement with 25 mg MGO incubated in 1 mL cell culture medium compared to bone cement without additive (control) in 1 mL (Figure 4A). Treatment with all other supernatants did not reduce viability. Proliferation of HOS cells (Figure 4B) was generally lower after treatment with the 1 mL supernatants compared to medium and the other supernatants. Bone cement with 25 mg MGO in 3 mL cell culture medium caused a significant reduction of HOS cell proliferation, but the 10 mL and 30 mL supernatants did not cause a reduction of proliferation. Release of IL-6 (Figure 4C) in the samples was only detected at low levels of less than 12 pg/mL, with no significant differences between the samples. High IL-6 release was only detected in the positive controls treated with LPS and PGN. Similar results were observed for the release of IL-1β (Figure 4D), with no significant differences between the samples and release of less than 10 pg/mL from all samples.

**FIGURE 5:**
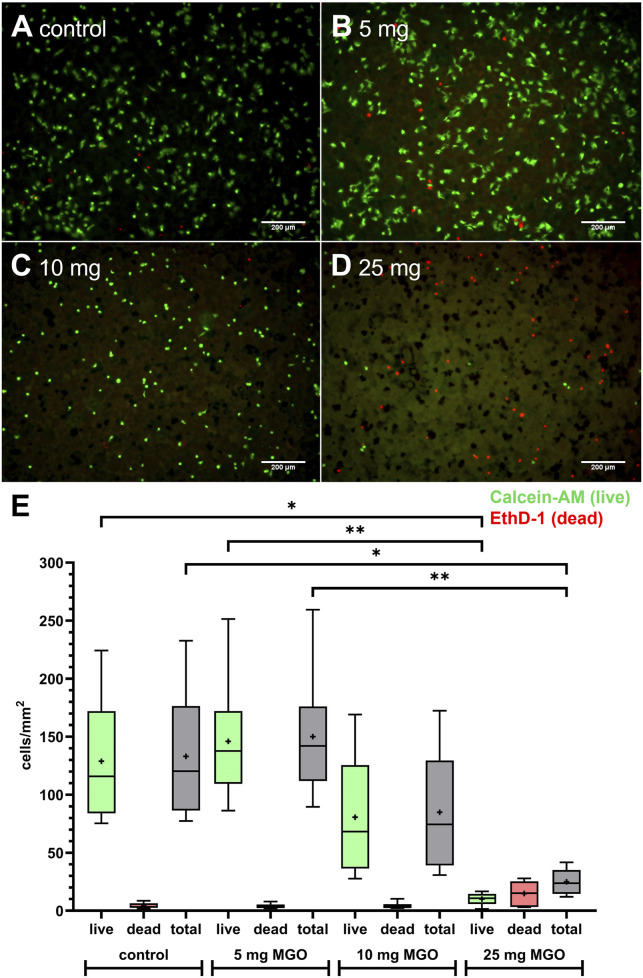
Growth and viability of HOS cells on bone cement platelets containing MGO or without additive. **(A**–**D)** Live/Dead staining of HOS cells seeded on bone cement platelets without additive **(A)** control), 5 mg MGO **(B)**, 10 mg MGO **(C)**, and 25 mg MGO **(D)**. Live cells are stained in green with Calcein-AM, dead cells are stained in red with ethidium homodimer-1 (EthD-1). Scale bar: 200 µm. **(E)** Live and dead cells per mm^2^ on bone cement platelets with MGO or without additive (n = 6 for each concentration). Data are shown as boxplots with min/max-whiskers and the mean depicted as +. Friedman test with Dunn’s multiple comparisons test, *p ≤ 0.05, **p ≤ 0.01.

HOS cells were seeded onto bone cement platelets to test growth and viability in direct contact (Figure 5). Cells were able to attach to the cement surface and were mostly viable (green cells, stained with calcein-AM) with only few dead cells (red, stained with ethidium homodimer-1 (EthD-1)) on bone cement without additive (Figure 5A). On bone cement with 5 mg MGO, the picture was similar compared to bone cement without additives with live cells in their usual stretched morphology and few dead cells (Figure 5B). With 10 mg MGO, fewer cells were attached to the cement surface but most were viable (Figure 5C), while with 25 mg MGO, most of the attached cells were dead (Figure 5D). Figure 5E shows the number of live and dead cells and the total cell number per mm^2^ on the bone cement. Although the effect was not significant, the number of live cells and the total cell number was reduced on bone cement with 10 mg MGO. The addition of 25 mg MGO to the bone cement reduced the number of live cells per mm^2^ significantly compared to the control and bone cement with 5 mg MGO. Along with that, the total number of cells per mm^2^ was also significantly lower compared to control and 5 mg MGO. Therefore, MGO reduced the attachment, growth and viability of HOS cells at MGO contents higher than 5 mg.

### 3.4 p38 MAPK activation

**FIGURE 6:**
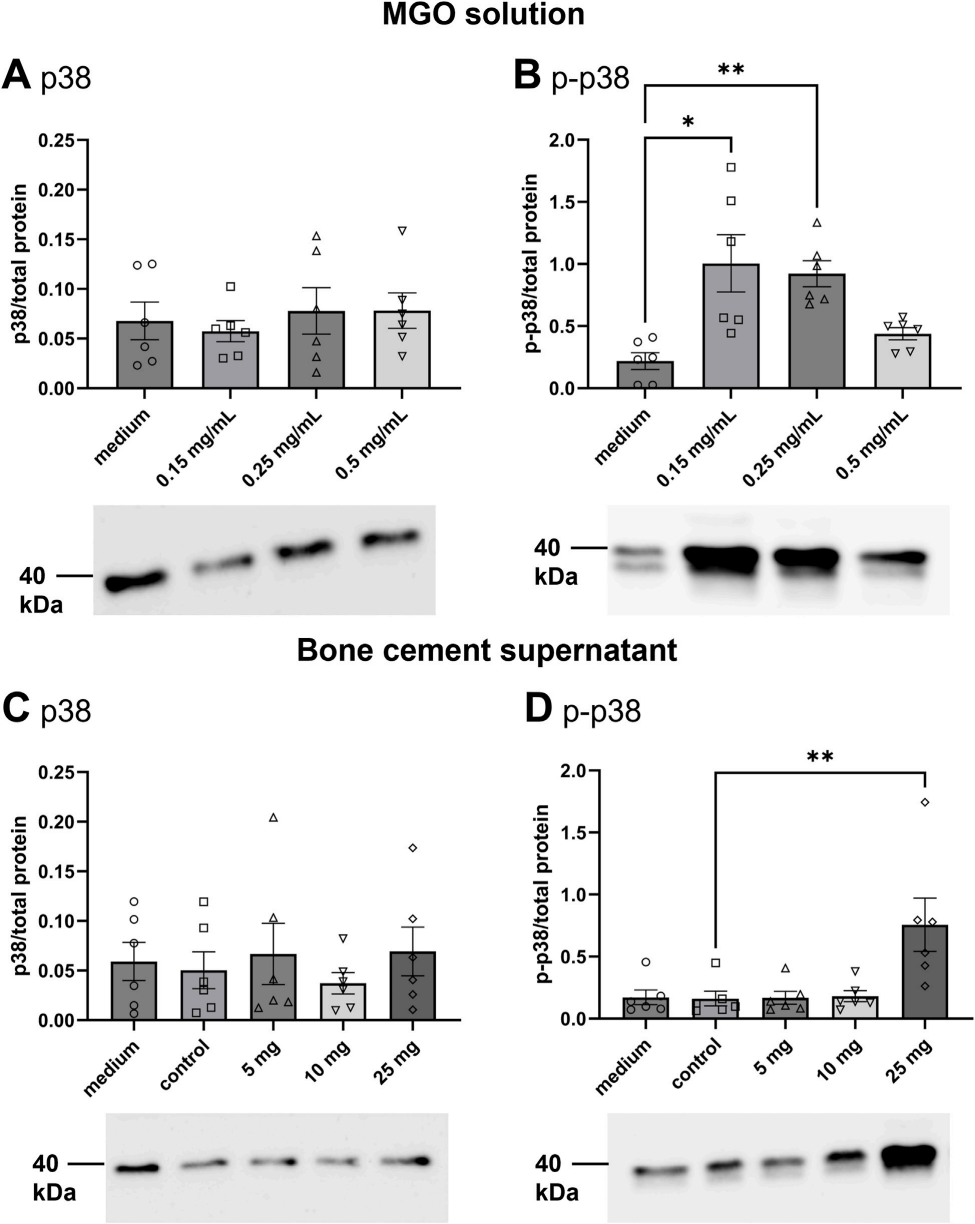
Detection of p38 and p-p38 in HOS cells treated with MGO solution or bone cement supernatants containing MGO. Graphs display protein band intensities normalized to total protein; photos show representative western blots. Protein size is expressed in kDa. **(A**,**B)** Levels of p38 **(A)** and p-p38 **(B)** and representative bands after treatment of HOS cells with 0.15 mg/mL, 0.25 mg/mL, 0.5 mg/mL MGO in cell culture medium or cell culture medium only. **(C**,**D)** Levels of p38 **(C)** and p-p38 **(D)** and representative bands after treatment of HOS cells with supernatants of bone cement with 5 mg MGO, 10 mg MGO, 25 mg MGO, without additive (control), or with cell culture medium. All data are shown as mean (n = 6) ± standard error of the mean (SEM). Individual data points are shown. Friedman test with Dunn’s multiple comparisons test, *p ≤ 0.05, **p ≤ 0.01.

Levels of p38 and its phosphorylated form p-p38 in HOS cells were analyzed after treatment with MGO solution or supernatants of bone cement with MGO (Figure 6). After treatment with MGO solution or cell culture medium, levels of p38 did not differ significantly (Figure 6A). The abundance of p-p38 was significantly higher after treatment with 0.15 mg/mL and 0.25 mg/mL MGO compared to cell culture medium (Figure 6B). After treatment with bone cement supernatants, the levels of p38 in HOS cells did not differ significantly (Figure 6C). Treatment with the supernatant of bone cement with 25 mg MGO elevated the level of p-p38 significantly. In conclusion, the contact of HOS cells with MGO led to phosphorylation and activation of p38.

## 4 Discussion

Despite the use of antibiotics with bone cement, PJIs are one of the main causes for implant failure. The global threat of antibiotic resistances makes it necessary to identify new or repurposed antibiotic effectors also for prevention and treatment of PJIs. Here, the effectivity of MGO as an antibacterial additive to bone cement and its biocompatibility was investigated. MGO inhibited growth of *S. pseudintermedius* at 0.15 mg/mL and reduced adherence and internalization of the bacteria into HOS cells at 0.05 mg/mL (Figure 2). Addition of 25 mg MGO to bone cement reduced bacterial growth on the bone cement surface (Figure 1). Below 0.15 mg/mL, MGO did not reduce HOS cell viability, but impaired proliferation (Figure 3). On bone cement, 10 mg and 25 mg MGO reduced attachment and growth of HOS cells (Figure 5) and 1 mL and 3 mL supernatants of bone cement with 25 mg MGO impaired cell viability and proliferation but did not cause release of IL-6 (Figure 4). Contact with MGO led to increased levels of p-p38 (Figure 6). Therefore, antibacterial effectiveness of MGO could be shown, but its biocompatibility was limited. Still, at non-cytotoxic concentrations, infection of HOS cells could be reduced.

For testing of antibacterial activity, *S. pseudintermedius* was chosen for this study because of its relevance for post-surgical infections in small animals (Hayes et al., 2013) and its increasing zoonotic potential (Van Hoovels et al., 2006; Vines et al., 2024). The MIC of 0.15 mg/mL determined for the three isolates of *S. pseudintermedius* used here is in the same range of MICs for other pathogens relevant to PJIs. While 0.079 mg/mL MGO inhibited growth of *S. aureus* and *E. coli* (Mavric et al., 2008) and 0.057 mg/mL inhibited growth of *Bacillus subtilis* (Rabie et al., 2016), the MICs for multiresistant *P. aeruginosa* ranged between 0.128 and 0.512 mg/mL (Hayashi et al., 2014). The formation of biofilms is a major virulence factor of *Staphylococci* and the majority of *S. pseudintermedius* can form biofilms (Singh et al., 2013). Biofilms of *S. pseudintermedius* consist of bacterial aggregates and microcolonies and present a heterogenous structure (Pompilio et al., 2015). In this study, multiresistant *S. pseudintermedius* aggregated and attached to the surface of bone cement platelets except for platelets supplemented with 25 mg MGO (Figure 1), showing that criteria for biofilms are fulfilled and that addition of 25 mg MGO can inhibit biofilm formation of *S. pseudintermedius*. Compared to other biofilm establishment studies, we used a relatively low inoculum for the growth of bacteria on bone cement with a 10^−6^-dilution of 0.5 McFarland standard, corresponding to 50 cfu/mL for *S. pseudintermedius*. For biofilm establishment on membrane filters and canine skin explants, an inoculum of 10^6^ cfu/mL was used (Kher et al., 2023), while Pompilio et al. (2015) established biofilms with 10^7^ cfu/mL *S. pseudintermedius*. A higher inoculum could lead to clearer results, however, only a low bacterial load is needed to initiate PJI in the presence of implant material (Zimmerli et al., 1982). It should also be considered that a possible mechanism for biofilm prevention is that MGO diffuses out of the bone cement into the growth medium, where it kills bacteria before they can attach. A possible way to reduce the necessary concentrations of MGO or antibiotics and to increase treatment success could be a combinational therapy with MGO and antibiotic drugs, also in bone cement. MGO can enhance the effectiveness of antibiotics (Mukherjee et al., 2011; Hayes et al., 2018). The ability of *S. pseudintermedius* to attach to and invade non-professional phagocytes like osteoblasts has been proven (Maali et al., 2016). Here, a concentration of MGO below the MIC reduced adherence and internalization of *S. pseudintermedius* (Figure 2). Pathomechanisms of *S.pseudintermedius* include binding to fibronectin with fibronectin-binding proteins on the bacterial surface to adhere and be internalized (Maali et al., 2016) and the release of pore-forming bacterial toxins (Maali et al., 2018). Formation of AGEs, interfering with gene expression and binding to fimbriae and flagellar proteins are the main antibacterial mechanisms of MGO (Rabie et al., 2016). These mechanisms can lead to the loss of function of surface proteins or a reduced ability to express toxins, which in turn could reduce the ability to adhere to and invade mammalian cells.

To ensure biocompatibility of MGO as an additive, experiments concerning the cytotoxicity and stress mechanisms have been conducted. Cytotoxicity of MGO is mainly based on the formation of reactive oxygen species (ROS) (Suh et al., 2016), furthermore, it can cause apoptosis (Liu et al., 2003). With 0.17 mg/mL, the IC_50_ for viability of HOS cells was above the concentration that reduced infection and just above the MIC (Figure 3A). In comparison, RAW264.7 macrophages tolerated MGO only up to concentrations of 0.02 mg/mL (Lee et al., 2019). Proliferation of HOS cells (Figure 3B) was more sensitive towards treatment and resulted in a U-shaped dose-response curve, indicating a hormetic effect. In another study measuring osteoblast proliferation after treatment with MGO (Seto et al., 2025), a similar effect could not be observed. MGO reduced proliferation in a dose-dependent manner. The reasons for a rise of proliferation at higher MGO concentrations compared to intermediate concentrations remain to be elucidated by further studies, since the underlying mechanisms of hormesis are complex and can involve various cellular and molecular pathways, including stress response pathways, antioxidant defense mechanisms, and DNA repair systems (Calabrese and Baldwin, 2002). Bone cement platelets with MGO were incubated with four different volumes of cell culture medium to simulate flow of tissue fluid around the implant and dilution of substance released from bone cement. Most substance is released from bone cement in the first hours after implantation, so that this early phase is critical for the viability and proliferation of implant-surrounding cells. Viability and proliferation of HOS cells was reduced by the higher concentrated supernatants of bone cement with MGO (Figure 4A,B), and proliferation was again more sensitive towards treatment. This shows that high MGO concentrations in bone cement could have a negative impact on post-surgical regeneration in differentiated cells, while proliferating cells can be stimulated by high MGO-concentrations. It was also analyzed if treatment with the MGO-containing supernatants would cause sterile inflammation and release of proinflammatory IL-6. In diabetic conditions, MGO causes elevated levels of IL-6 and other cytokines such as IL-1β and therefore inflammation in patients, contributing to the pathology of diabetes. Patients with elevated levels of IL-6 and IL-1β have a higher risk of even developing diabetes (Alexandraki et al., 2006). Treatment of osteoblasts with MGO for 48 h led to increased release of IL-6 (Suh et al., 2015). Interestingly, similar results could not be shown in this study because treatment with MGO-containing supernatants did not cause increased IL-6 release compared to control (Figure 4C). IL-1β is a proinflammatory cytokine and a strong stimulator of bone resorption by stimulating osteoclastogenesis (Ruscitti et al., 2015). Here, treatment with the MGO-containing bone cement supernatants did not lead to elevated IL-1β release (Figure 4D). One reason for this may be the simultaneous release of bone cement ingredients in the present study that inhibit IL-6 and IL-1β release by MGO. The composition of the bone cement surface affects which proteins can bind, how cells attach and therefore impact growth of surrounding cells for integration of the implant (Jiang et al., 2025). MGO-binding proteins could therefore improve cellular attachment. Therefore, HOS cells were also seeded directly onto the bone cement platelets and their attachment and viability was analysed (Figure 5). Addition of 25 mg MGO to the bone cement reduced attachment and viability of HOS cells significantly, which is in line with the results from treatment with bone cement supernatants. Reduced attachment of HOS cells was also observed on bone cement with 10 mg MGO. Since MGO is also released into the cell culture medium, it is possible that MGO has a cytotoxic effect on cells before they can attach. For regeneration after surgery, it is also important to keep in mind that MGO can impair osteoblast differentiation and mineralization in addition to reducing viability (Seto et al., 2025). A pathway involved in cellular stress reaction is the pathway of p38 mitogen-activated protein kinase (MAPK). P38 is activated through different stress stimuli, e.g., UV radiation, heat, or oxidative stress and is responsible for downstream regulation of cell cycle, apoptosis, and inflammation (Zarubin and Han, 2005). For activation, p38 is phosphorylated (phospho-p38 or p-p38). In rat mesangial cells, MGO treatment caused apoptosis through the activation of p38 in a dose-dependent manner (Liu et al., 2003). This effect was also observed in rat Schwann cells in the context of diabetic neuropathy (Fukunaga et al., 2005). In the bone, the activation of p38 is involved in the conversion of macrophages to osteoclasts after 6 h of treatment with MGO, leading to osteoporosis (Lee et al., 2019). The p38 pathway in HOS cells is often analyzed in the context of anti-tumor treatments. The pathway has been shown to be involved in the anti-tumor effect of deoxyshikonin (Hsieh et al., 2023) and Licochalcone A (Lin et al., 2019). In this study, we showed that locally given MGO lead to significant activation of p38, displaying a cellular stress reaction (Figure 6). Treatment with the supernatant of bone cement with 25 mg MGO caused increased levels of p-p38 and a reduction of viability and proliferation. The reduction of viability and proliferation could be due to apoptosis, mediated through the p38 MAPK pathway, but must be elucidated by further analysis, e.g., by caspase-3 measurements. Therefore, effective concentrations of MGO to be added to bone cement must be chosen wisely to avoid apoptosis and stress in cells surrounding the implant.

This study was not without limitations. The exact amount of MGO released from the platelets was not measured so that the exact concentrations in the supernatants are not known. In addition, including different types of bone cells into the experiments would have provided more knowledge about the interaction of MGO with bone tissue, especially since MGO can activate osteoclasts in diabetic conditions (Lee et al., 2019). Since old age and diabetes are risk factors not only for PJI but also for osteoporosis, the risk of osteoporosis caused by MGO needs to be addressed in follow-up research. Future research should also address development of resistance against MGO in PJI-relevant pathogens. Stability of bone cement with MGO was not considered here because the study aimed to evaluate MGO as an active substance. However, mechanical studies should be conducted in the further development of bone cement with MGO, since additives can decrease the mechanical stability of bone cement significantly (Arora et al., 2013). The heat stability of MGO should also be considered when using it as an additive to bone cement. PMMA bone cement reaches temperatures of more than 70 °C during polymerization and hardening (Szoradi et al., 2024), while MGO in Manuka honey is lost when heated to 90 °C (Kato et al., 2021). Encapsulation of MGO before addition to bone cement, e.g., in nanotubes (Shen et al., 2019), could help to shield MGO from the heat generated during cement polymerisation.

## 5 Conclusion

In the present study, MGO could effectively inhibit growth of PJI-relevant *S. pseudintermedius* isolates on the surface of bone cement and reduce the infection of HOS cells. At the MIC, MGO displayed low cytotoxicity. Incorporated into bone cement, MGO reduced cell viability and proliferation and impaired attachment to the surface at high concentrations, especially at the antibacterial concentration of 25 mg MGO per platelet. Increased release of IL-6 was not measured, but levels of p-p38 MAPK were elevated, which indicates that MGO treatment could lead to apoptosis. Taking these results together, further research is necessary to enhance the biocompatibility of MGO, since it represents a potential agent for biomedical devices.

## Data Availability

The raw data supporting the conclusions of this article will be made available by the authors, without undue reservation.
