# Supplementary material for: In vitro evaluation of methylglyoxal as an antibacterial additive to bone cement
Source: Front Bioeng Biotechnol. 2025 Sep 10;13:1661383. doi: 10.3389/fbioe.2025.1661383 (PMC12457313; doi:10.3389/fbioe.2025.1661383)
Supplement: Supplementary file 1 [file DataSheet1.pdf]

Supplementary Figure 1: Uncropped Western Blots

Figure 6A: p38; MGO in mg/mL

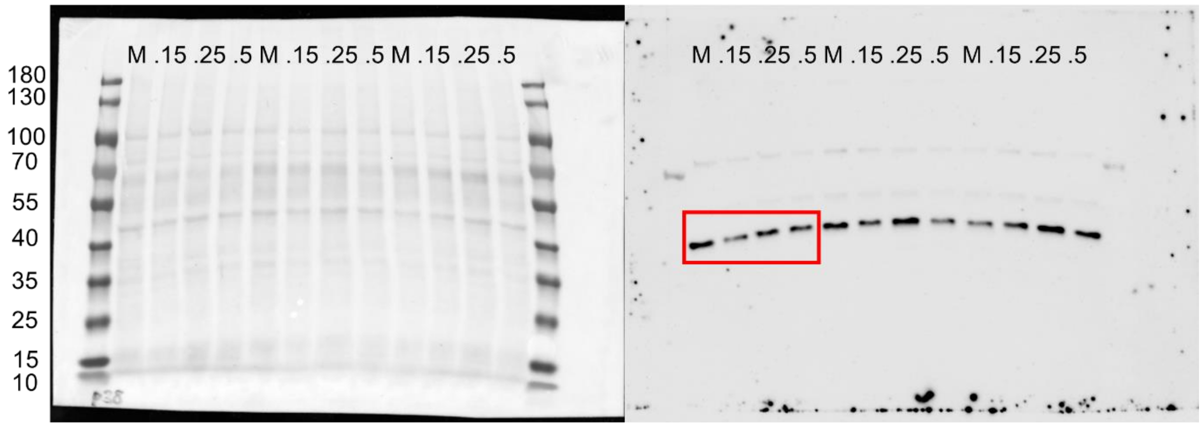

Figure 6B: p-p38, MGO in mg/mL

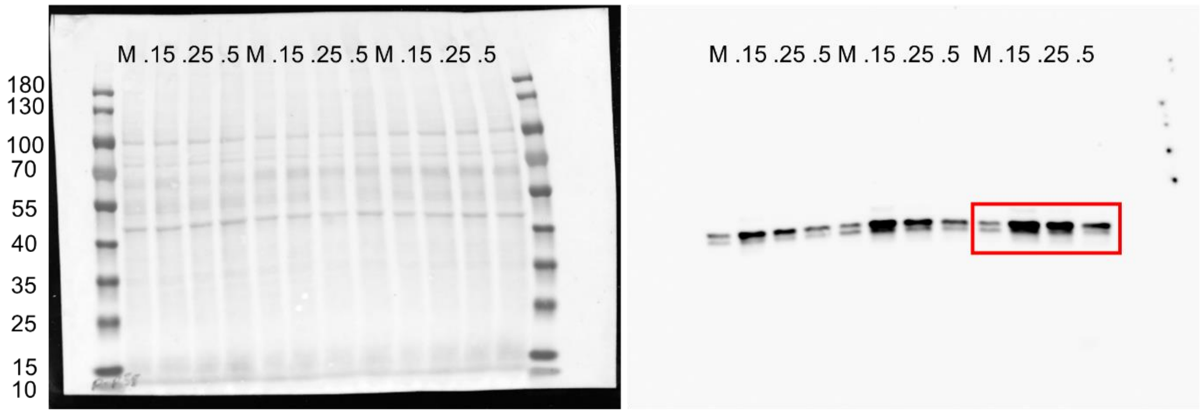

Figure 6C: p38, MGO in bone cement in mg

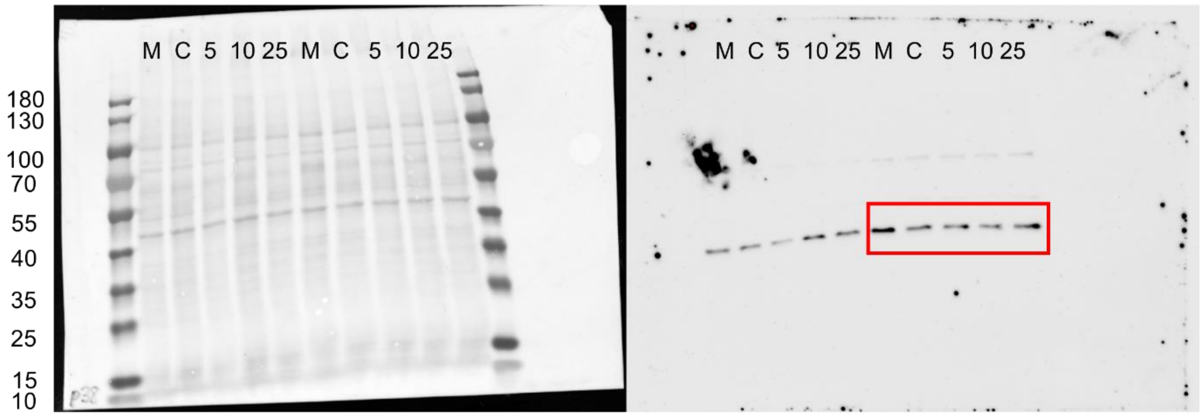

Figure 6D: p-p38, MGO in bone cement in mg

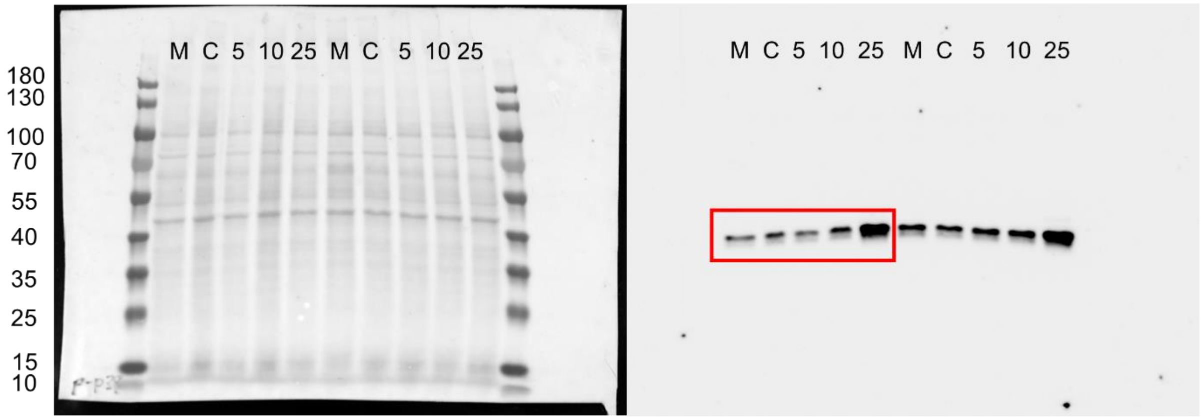

Figure S1: Full blots of cropped images shown in Figure 6. Left: visualized with Ponceau S red staining and used for normalization. Right: specific protein as indicated visualized with chemiluminescence. Protein size is expressed in kDa. Red boxes highlight the bands depicted in the manuscript. Concentrations of pure MGO are shown in mg/mL (0.15, 0.25, and 0.5 mg/mL), amount of MGO per bone cement platelet is shown in mg (5, 10 and 25 mg). M = medium; C = control, bone cement without additive.

Supplementary Figure 2: Live/Dead staining of bone cement platelets incubated without bacteria

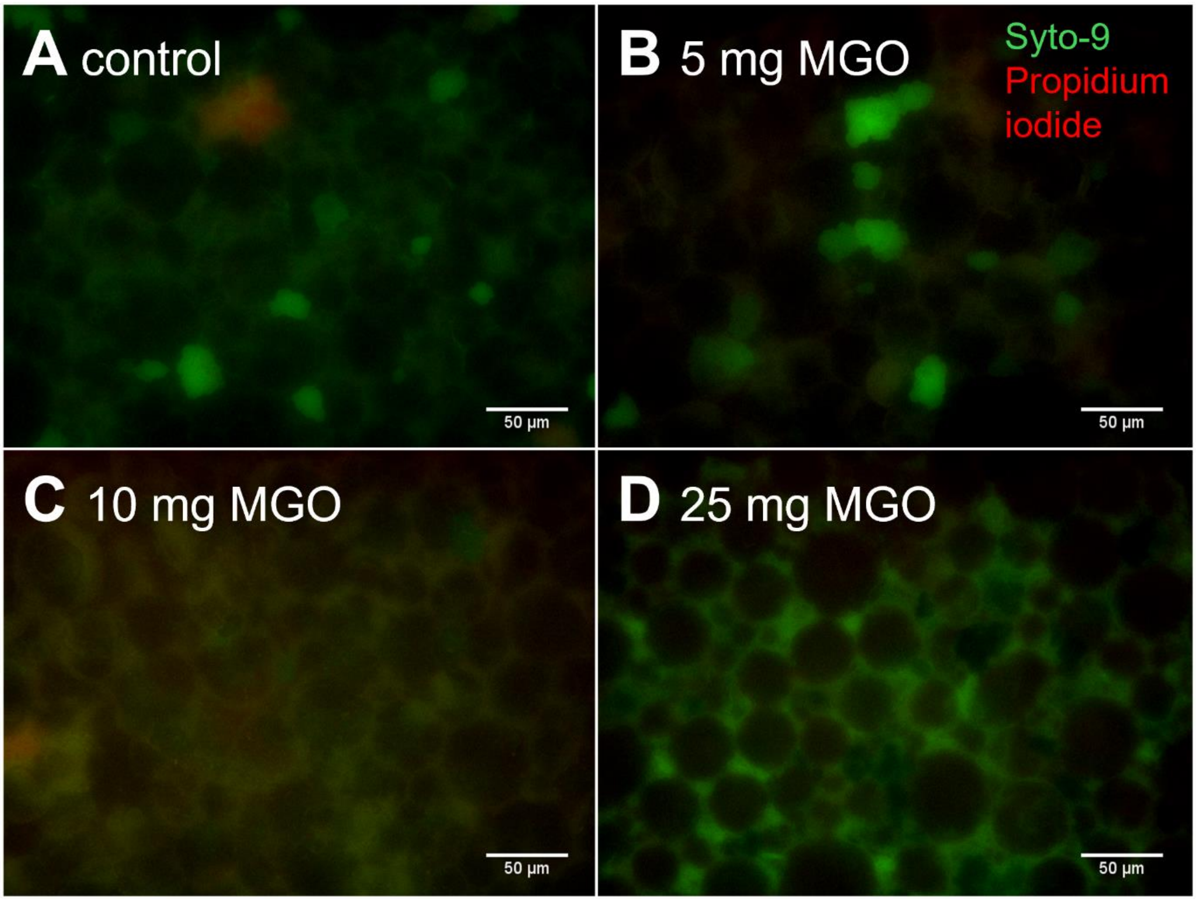

Figure S2: Overlay images of Live/Dead-stained bone cement platelets incubated without bacteria for imaging of background fluorescence. The images serve as controls for photos shown in Figure 1. Syto-9 (green) is a fluorescent dye for live cells, propidium iodide (red) dyes dead cells. **(A)**: Background fluorescence and artifacts on bone cement without additive. **(B)**: Background fluorescence and artifacts on bone cement with 5 mg MGO. **(C)**: Background fluorescence of bone cement with 10 mg MGO. **(D)**: Background fluorescence of bone cement with 25 mg MGO. Scale bar: 50 µm.
